# Transient receptor potential channel involvement in antinociceptive effect of citral in orofacial acute and chronic pain models

**DOI:** 10.17179/excli2022-5042

**Published:** 2022-06-24

**Authors:** Sacha Aubrey Alves Rodrigues Santos, Marina de Barros Mamede Vidal Damasceno, Francisco Ernani Alves Magalhães, Barry John Sessle, Breytiner Amaro de Oliveira, Francisco Lucas Alves Batista, Antônio Eufrásio Vieira-Neto, Adriana Rolim Campos

**Affiliations:** 1 Experimental Biology Center, University of Fortaleza, Fortaleza, Brazil; 2 Department of Nutrition and Health, State University of Ceará, Fortaleza, Brazil; 3 Department of Physiology and Faculty of Dentistry, University of Toronto, Toronto, Canada

**Keywords:** citral, TRP channels, orofacial nociception

## Abstract

This study aimed to test for the possible antinociceptive effect of the naturally occurring terpene citral in rodent models of acute and chronic orofacial pain and to test for the possible involvement of transient receptor potential (TRP) channels in this effect. Acute nociceptive behavior was induced in one series of experiments by administering formalin, cinnamaldehyde, menthol or capsaicin to the upper lip. Nociceptive behavior was assessed by orofacial rubbing, and the effects of pre-treatment with citral (0.1, 0.3 or 1.0 mg/Kg) or vehicle (control) were tested on the behavior. Nociceptive behavior was also induced by formalin injected into the temporomandibular joint or mustard oil injected into the masseter muscle, preceded by citral or vehicle (control) treatment. The chronic pain model involved infraorbital nerve transection (IONX) that induced mechanical hypersensitivity which was assessed by von Frey hair stimulation of the upper lip. Motor activity was also evaluated. Docking experiments were performed using TRPV1 and TRPM8 channels. Citral but not vehicle produced significant (p<0.01, ANOVA) antinociception on all the acute nociceptive behaviors, and these effects were attenuated by TRPV1 antagonist capsazepine, TRPM3 antagonist mefenamic acid and by TRPM8 desensitization, but not by ruthenium red and TRPA1 antagonist HC-030031. The IONX animals developed facial mechanical hypersensitivity that was significantly reduced by citral but not by vehicle. The docking experiments revealed that citral may interact with TRPV1 and TRPM8 channels. These results indicate the potential use of citral as an inhibitor of orofacial nociception in both acute and chronic pain states through TRPV1, TRPM3 and TRPM8 channels.

See also Figure 1(Fig. 1).

## Nomenclature and Abbreviations

ANOVA analysis of variance

C vehicle control

Ca^2+^ calcium

Capz capsazepine

HC HC-030031

ION infraorbital nerve

IONX infraorbital nerve transection

MA mefenamic acid

NaCl sodium chloride

NaHCO_3_ sodium bicarbonate

NaH₂PO₄ sodium phosphate

PBS phosphate-buffered saline

p.o. per os

RR ruthenium red

SEM standard error of mean

TMJ temporomandibular joint

TRP transient receptor potential

TRPA1 transient receptor potential ankyrin type 1

TRPM3 transient receptor potential melastatin type 3

TRPM8 transient receptor potential melastatin type 8

TRPV1 transient receptor potential vanilloid type 1

## Introduction

Orofacial pain may be associated with considerable suffering and disability of patients and can be challenging to diagnose and manage successfully. Appropriate management is reliant on in-depth knowledge of the pathophysiology of orofacial pain and the multidisciplinary therapeutic approaches that are available to control it (Romero-Reyes and Uyanik, 2014[57]; Sessle, 2014[61]; Gilkey and Plaza-Villegas, 2017[13]). This includes pharmacological treatment which nonetheless can be difficult since the diverse nature and effects of the pain require different pharmacological approaches (Weiss et al., 2017[68]; Heir, 2018[17]).

The transient receptor potential (TRP) ion channels have been the most studied proteins involved in pain in recent years, and the use of TRP channel modulation has been identified as an important strategy for the management of many pain conditions (Luo et al., 2021[38]; Hossain et al., 2019[19]; Lee et al., 2019[33]; Jansen et al., 2019[22]). The increase in expression of TRP channels, such as TRPV1 and TRPA1, that has been documented in the trigeminal system in animal models of orofacial pain suggests that modulation of these channels may indeed be an important strategy for the management of orofacial pain (Luo et al., 2021[38]; Hossain et al., 2019[19]; Lee et al., 2019[33]). The use of TRP channel modulation has been identified as an important strategy for management of many pain conditions (Jansen et al., 2019[22]). TRP channels participate in the control of cell influx of Ca^2+^ (Gess et al., 2010[12]) and while their structural diversity may result in their participation in different signaling processes, this diversity has impaired the development of safe and effective modulators (Moore et al., 2018[47]). It has been reported that the combined development of modulators of specific TRP channels can be an alternative to the direct inhibition of each channel (Hossain et al., 2019[19]; Akopian, 2011[1]; Giorgi et al., 2019[14]). The use of naturally occurring and synthetic terpenes is one such possible approach. They stimulate TRP channel-mediated signaling in mammals, and some of these signaling molecules can act on several different TRP channel protein targets (Janero and Makriyannis, 2014[21]). 

Citral (3,7-dimethyl-2,6-octadienal) is an open-chain monoterpenoid and a mixture of two isomers (neral *cis*-isomer and geranial *trans*-isomer) and is present in the essential oils of various medicinal plants such as *Cymbopogon citratus* (Nishijima et al., 2014[50]). Citral has been shown to act as a partial TRPV1, TRPV2, TRPV3, TRPM8, and TRPA1 channel agonist, and prolonged inhibition of TRPV1, TRPV2, TRPV3 and TRPM8 following their activation is considered an important pharmacological effect of this monoterpene (Stotz et al., 2008[62]). The anti-hyperalgesic and anti-inflammatory effect of citral has been previously reported (Nishijima et al., 2014[50]; Quintans-Júnior et al., 2011[55]; Campos et al., 2019[4]; Gonçalves et al., 2020[15]), but its effect on nociceptive processes underlying acute and chronic orofacial pain has not yet been investigated. Therefore, this study aimed to test for the possible antinociceptive effect of citral in rodent models of acute and chronic orofacial pain and to test for the possible involvement of TRP channels in this effect.

## Materials and Methods

### Drugs and reagents 

The following drugs and reagents were used in the study: citral which was dissolved in 0.25 % Tween 80 and purchased from Sigma-Aldrich (USA); glacial acetic acid, sodium chloride and Tween 80 which were purchased from Dinâmica^®^ (Brazil); formaldehyde which was purchased from Vetec^®^ (Brazil); saline solution (0.9 %) which was purchased from Arboreto^®^ (Brazil); ketamine and xylazine which were purchased from Syntec^® ^(Brazil); capsaicin, cinnamaldehyde, glutamate, mefenamic acid, menthol, capsazepine, ruthenium red, mustard oil and HC-030031 which were purchased from Sigma-Aldrich (USA). In addition, phosphate-buffered saline (PBS) was prepared using 0.15 M NaCl (Cromoline®, Brazil), 0.01 M NaH₂PO₄ (Vetec^®^, Brazil) and NaCOH_3_ (Vetec^®^, Brazil; *quantum sufficit* to pH 7.2).

### Animals

Swiss mice (20-30 g) and Wistar rats (200-250 g), which were 8-weeks old, were obtained from the Núcleo de Biologia Experimental (NUBEX) of the Universidade de Fortaleza (UNIFOR), Brazil. The specific-pathogen-free animals were housed in appropriate cages (IVC cages, Techniplast®) and kept at a room temperature of 22-24 °C on a 12:12 h light: dark cycle. They received standard feed (Purina, São Paulo, Brazil) and water *ad libitum*. All protocols were in strict compliance with the standards established by Brazil's National Council on Animal Experimentation Control and received approval from the Committee on Animal Research and Ethics of UNIFOR (#005/2017). To follow animal ethical guidelines for conserving on the number of animals needed to meet study objectives, only male animals were used in this study (Guidelines for the Use of Animals, 2012[16]).

The tests of cutaneous orofacial nociception are described below; as well as the rotarod test can be performed on mice and rats, but for reasons of economy, we decided to use mice for these tests in this study. The models of temporomandibular nociception, craniofacial nociception and infraorbital nerve transection (see below) are typically used in rats, and therefore, rats were used in this part of the study.

### Treatments

For each experiment, animals (n=6/ group) were divided into the following groups:

**1 - Vehicle control** (*per os - orally; p.o.*; 0.9 % NaCl + 0.25 % Tween 80).

**2 - Previous studies used citral at doses of 25, 100 and 300 mg/Kg (*p.o.*)** (Nishijima et al., 2014[50]; Mota et al., 2020[48]). Here, in preliminary experiments, eight doses of citral (0.1, 0.3, 1.0, 3.0, 10.0, 30.0, 100 and 300 mg/Kg; *p.o.*) were examined in the formalin test (see below). All doses produced an antinociceptive effect (****p <0.001 *vs *control; Table 1(Tab. 1)). Consequently, 0.1, 0.3 and 1.0 mg/Kg (*p.o.)* were chosen as the final doses for the cinnamaldehyde, menthol and capsaicin tests. Since there was no difference in effects between doses (Tables 1(Tab. 1) and 2(Tab. 2)), citral at 0.1 mg/Kg (*p.o.)* was used for all other tests. With a view to clinical translation of the findings to pain management, the oral route was used in the present study since this is the route associated with enhanced therapeutic benefits due to the increase in patient compliance with this route of drug administration (Chenthamara et al., 2019[6]; Homayun et al., 2019[18]; Jin et al., 2008[24]).

**3 - Naïve (no treatment)**.

### Antinociceptive activity

#### Formalin-induced orofacial nociception 

Mice (n=6/group) received 2 % formalin (TRPA1 agonist, 20 μL, s.c) injected with a 27-gauge needle into the right upper lip (perinasal area). Nociception was quantified as the time the mouse spent rubbing the site of injection with its fore- or hind-paw at 0-5 minutes (1^st^ phase) and at 15-30 minutes (2^nd^ phase) after the injection, in accordance with a previous study (Luccarini et al., 2006[37]). To assess the effect of the test drug, mice were pre-treated (10 mL/Kg; *p.o.*) with vehicle (control) or citral (0.1, 0.3, 1.0, 3.0, 10.0, 30.0, 100 and 300 mg/Kg; *p.o.*) 60 minutes before the formalin injection. A naïve group (n=6) was also included.

#### Cinnamaldehyde-induced orofacial nociception 

Mice (n=6/group) received cinnamaldehyde (TRPA1 agonist specific, 0.66 μM, 20 μL (Nomura et al., 2013[51]), injected with a 27-gauge needle into the right upper lip (perinasal area). Nociception was quantified as the time the mouse spent rubbing the site of injection with the fore- or hind-paw 0-5 minutes after the injection of cinnamaldehyde, in accordance with a previous study (Nomura et al., 2013[51]). Citral (0.1, 0.3 and 1.0 mg/Kg; *p.o*.) and vehicle (10 mL/Kg; *p.o*.)were administered 60 minutes before the cinnamaldehyde injection. A naïve group (n=6) was also included.

In subsequent experiments, different groups of mice (n=6/each) were pretreated by subcutaneous injection into the upper lip (perinasal area) of the antagonist HC-030031 (TRPA1 antagonist (Eid et al., 2008[11]); 20 μL, 0.1 mg/mL) 15 minutes prior to administration of citral (0.1 mg/Kg; *p.o.*), in accordance with a previous study (Banzawa et al., 2004[3]) to check for their possible effects on TRPA1 channels. 

Since citral has effects on several TRP channels (Stotz et al., 2008[62]), mefenamic acid (TRPM3 antagonist (Klose et al., 2011[27]); 20 μL, 30 mM) was administered 15 minutes prior to administration of citral (0.1 mg/Kg; *p.o.*) to check for the possible involvement of TRPM3 channels that may have contributed to the effects of citral. 

#### Menthol-induced orofacial nociception 

Mice (n=6/group) received menthol (TRPM8 agonist (Macpherson et al., 2006[41]); 20 μL, 1.2 mM) through a 27-gauge needle into the right upper lip (perinasal area). Nociception was quantified as the time the mouse spent rubbing the site of injection with the fore- or hind-paw 0-10 minutes after the injection, in accordance with a previous study (Liu et al., 2013[35]). Citral (0.1, 0.3 and 1.0 mg/Kg; *p.o.*) and vehicle (10 mL/Kg; *p.o*.) were administered 60 minutes before the cinnamaldehyde injection. A naïve group (n=6) was also included.

The antagonist (AMTB) was not available to us, so we decided to use desensitization with menthol to test the involvement of TRPM8. In a subsequent experiment, we used high doses of menthol to desensitize the TRPM8 channels in other groups of animals. The four groups of animals (n=6/each) were:

a) Vehicle control (*per os* - *p.o*.; 0.9 % NaCl + 0.25 % Tween 80); 

b) Desensitized - Menthol (1.2 mM; 20 μL; intraperitoneal; i.p.) during 15, 30, 60, 120 and 240 minutes prior to vehicle control;

c) Citral (0.1 mg/Kg; *p.o*.);

d) Desensitized + Citral - Desensitized with menthol (as above) prior to citral.

Orofacial nociception was induced by capsaicin 1 hour after the treatments (a-d). A naïve group (n=6) was included. The nociceptive behavior was observed for each animal during 10-20 minutes.

#### Capsaicin-induced orofacial nociception

Mice (n=6/group) received capsaicin (TRPV1 agonist (Pelisser et al., 2002[54]), 2.5 μg, dissolved in ethanol, PBS, and distilled water in a 1:1:8 ratio) injected with a 27-gauge needle into the right upper lip (perinasal area). Nociception was quantified as the time the mouse spent rubbing the site of injection with the fore- or hind-paw 10-20 minutes after the injection of capsaicin in accordance with a previous study (Pelisser et al., 2002[54]). Citral (0.1, 0.3 and 1.0 mg/Kg; *p.o.*) and vehicle (10 mL/Kg; *p.o*.) were administered 60 minutes before the cinnamaldehyde injection. A naïve group (n=6) was also included.

In subsequent experiments, mice (n=6/ group) were pretreated by subcutaneous injection into the upper lip (perinasal area) of the antagonist ruthenium red (non-selective TRP antagonist (Clapham et al., 2005[7]); 20 μL, 10 nM) or capsazepine (competitive TRPV1 antagonist (Ducrocq et al., 2019[9]); 20 μL, 30 nM), 15 minutes prior to administration of citral (0.1 mg/Kg; *p.o*.), in accordance with a previous study (Maia et al., 2006[42]) to check for their possible effects on the TRPV1 channel that may have contributed to the effects of citral. 

#### Formalin temporomandibular joint (TMJ) nociception 

Rats (n=6/group) were acclimated individually in a glass test chamber (30 x 30 x 30 cm) for 30 minutes to minimize stress. The animals were pre-treated (10 mL/Kg; *p.o*.) with vehicle or citral (0.1 mg/Kg; *p.o*.) and 60 minutes later, the left TMJ was injected with 50 μL 2.0 % formalin by a Hamilton syringe and a 30-gauge needle, in accordance with a previous study (Roveroni et al., 2001[58]). A sham group (n = 6) receiving 0.9 % NaCl (50 μL) and a naïve group (n=6) were also included.

The animals were returned individually to the test chamber to quantify nociception as rubbing of the orofacial region with the ipsilateral fore- or hind-paw, and head flinching (intermittent and reflexive shaking of the head) and chewing (Roveroni et al., 2001[58]). The time that the rat spent rubbing the orofacial region was registered at 3 minutes blocks for 36 minutes. Head flinching and chewing were registered by their absence or presence.

#### Mustard oil-induced craniofacial nociception 

Rats (n=6/group) were acclimated individually in a glass test chamber (30 x 30 x 30 cm) for 30 minutes to minimize stress. The rats were pre-treated (10 mL/Kg; *p.o*.) with vehicle or citral (0.1 mg/Kg; *p.o*.) 60 minutes before the injection into the left masseter muscle of mustard oil (20 %; 20 μL) in a Hamilton syringe and a 30-gauge needle. Sham and naïve groups (n=6/each) were also included. The ipsilateral hind-paw shaking was quantified by counting the number of paw shakes in 30 seconds intervals during 4 minutes, in accordance with a previous study (Ro et al., 2003[56]).

#### Assessment of mechanical sensitivity after infraorbital nerve transection 

Rats (n=6/group) were anesthetized with ketamine (100 mg/Kg; i.p.) and xylazine (10 mg/Kg; i.p.) and the left infraorbital nerve (ION) was exposed at its entry into the infra-orbital foramen by way of an intra-oral incision (2 mm) in the oral mucosa of the left fronto-lateral maxillary vestibulum, as previously described by Kumar et al. (2013[30]). The ION was lifted from the maxillary bone and cut (IONX) without damaging adjacent nerves and vessels. Subsequently, the animals were returned to their cages and fed with mash and chow. The animals were monitored daily in the post-operative period. Rats were divided into four groups: citral (0.1 mg/Kg; *p.o*.); vehicle; naïve and sham-operated animals (n=6/each group).

The rats were acclimated, accustomed, and tested for facial mechanical sensitivity one day prior to nerve transection (baseline) and on postoperative days 1, 3, 5, 7, 10, 14 and 21, as previously described (Kumar et al., 2013[30]). A single dose (10 mL/Kg; *p.o*.) of citral or vehicle was administered by gavage at each postoperative day, and mechanical sensitivity of the left whisker pad skin was assessed using von Frey hairs. The head-withdrawal threshold to mechanical stimulation of the whisker pad skin was defined as the minimum force needed to evoke an escape more than 3 times as a result of 5 stimuli.

#### Molecular docking study - TRPV1 and TRPM8

The interaction between citral and TRPV1 was analyzed *in silico* by using molecular docking simulation, which consists of the use of a computational algorithm with a specific software for coupling two molecules, seeking to form a stable complex. The three-dimensional structures of neral and geranial (citral isomers) and TRPV1 and TRPM8 channels were obtained in PubChem and Protein Data Bank (643779, 638011 and 3J5P, respectively). To validate the simulation, the TRPV1 channel was also docked against the already established TRPV1 antagonist capsazepine (PubChem ID: 2733484). The docking was performed using the software: HEX 8.0.0 (Macindoe et al., 2010[40]), which performs the docking automatically, seeking all the possible binding sites based on the energy (i.e., strength) of association between citral isomers and the TRPV1 and TRPM8 channels in certain positions. The provided data was analyzed using PyMol v1.4.7.

#### Rotarod test

To rule out citral effects on motor coordination that may confound assessment of nociceptive behavior, mice (n=6/group) were selected 24 hours previously by eliminating animals unable to remain on the rotarod for a period of 60 seconds. The animals were treated (10 mL/Kg; *p.o.*) with vehicle or citral (0.1, 0.3 and 1.0 mg/Kg; *p.o*.) 60 minutes prior to placement on the rotating rod. The latency to falling was measured for up to 60 seconds. Results were expressed as average time in seconds spent on the rod in each group (Dunham and Miya, 1957[10]). 

#### Statistical analyses 

The results are presented as mean ± S.E.M. values of each group of 6 animals. Normality of the distribution was confirmed (Kolmogorov-Smirnov) and group data were submitted to an analysis of variance (one-way ANOVA) followed by the Tukey *post-hoc* test or to a two-way ANOVA followed by Bonferroni´s post-hoc test. For analyses of citral (and vehicle) effects in IONX animals, the baseline was normalized and all values post-citral or post-vehicle administration were expressed as percentage (%) change from baseline. The level of statistical significance was set at 5 % (p < 0.05).

## Results

### Antinociceptive effect of citral

Formalin injection into the upper lip of mice increased the rubbing of the face (Table 1(Tab. 1)), and citral (0.1, 0.3, 1.0, 3.0, 10.0, 30.0, 100 and 300 mg/Kg) reduced (p<0.0001) the face rubbing induced by formalin when compared with vehicle control in both phases (1^st^ phase: 0-5 minutes and 2^nd^ phase: 15-30 minutes) of the test (Table 1(Tab. 1)). There was no difference between the doses and 0.1, 0.3 and 1.0 mg/Kg were chosen as the final doses for further experiments using cinnamaldehyde, capsaicin or menthol.

Cinnamaldehyde, capsaicin or menthol injection into the upper lip of mice also increased the face rubbing that was analyzed during the following post-injection time periods (0-5 minutes cinnamaldehyde; 0-10 minutes capsaicin; and 10-20 minutes menthol). Pre-treatment with citral (0.1, 0.3 and 1.0 mg/Kg) was associated with a significant reduction in the face rubbing induced by cinnamaldehyde, capsaicin and menthol when compared with vehicle control (p<0.0001; Table 2(Tab. 2)). There was no difference between citral and naïve groups (Table 2(Tab. 2)).

**Figure 2:**
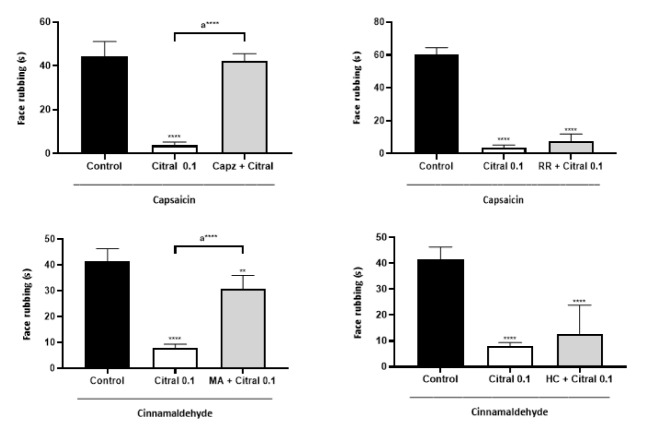
Effect of capsazepine (Capz; upper left panel), ruthenium red (RR; upper right panel), mefenamic acid (MA; lower left panel) and HC-030031 (HC; lower right panel) on the antinociceptive activity of citral (0.1 mg/Kg) in the orofacial nociception models reflected in capsaicin- or cinnamaldehyde-induced face rubbing in mice. Each column represents the mean ± S.E.M (n = 6/group). Tukey's test (*p<0.05, ***p < 0.001, ****p < 0.0001 vs control and a ****p < 0.0001 vs Citral 0.1). Capsaicin (2.5 µg), capsazepine (30 nM), ruthenium red (10 nM), cinnamaldehyde (0.66 µM), HC-030031 and (0.1 mg/mL) mefenamic acid (30 nM) were injected into the upper lip of animals 60 minutes after citral or vehicle (control) administration. Citral and vehicle (control) were administered by gavage.

**Figure 3:**
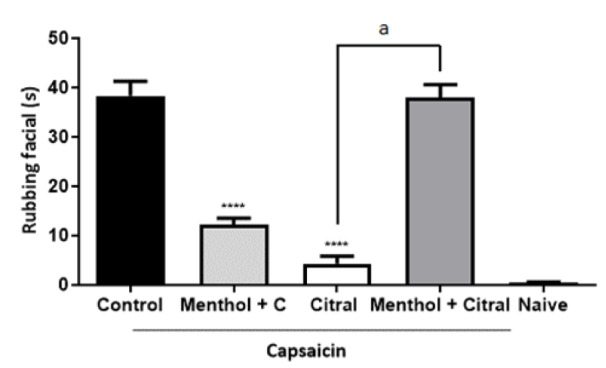
Involvement of the TRPM8 in the antinociceptive effect of citral (0.1 mg/Kg) in mice. Each column represents the mean ± S.E.M (n = 6/group). One-way ANOVA with *post-hoc* Tukey's test (****p<0.0001* vs* control and ^a^p<0.0001 *vs* Citral). C - vehicle control. Menthol (1.2 mM) was injected (intraperitoneal) 15, 30, 60, 120 and 240 minutes prior to citral or vehicle (control). Capsaicin (2.5 µg) was injected into the upper lip of animals 60 minutes after citral or vehicle (control) administration. Citral and vehicle (control) were administered by gavage.

In the capsaicin test, the decreased in face rubbing induced by citral was abolished (Figure 2(Fig. 2)) by pretreatment with capsazepine and mefenamic acid, but not by ruthenium red and HC-030031 (Figure 2(Fig. 2)). The desensitization of TRPM8 channels by menthol decreased the capsaicin-induced face rubbing (p < 0.001 vs control) and abolished the decreased in face rubbing induced by citral (0.1 mg/mL; Figure 3(Fig. 3)).

**Figure 4:**
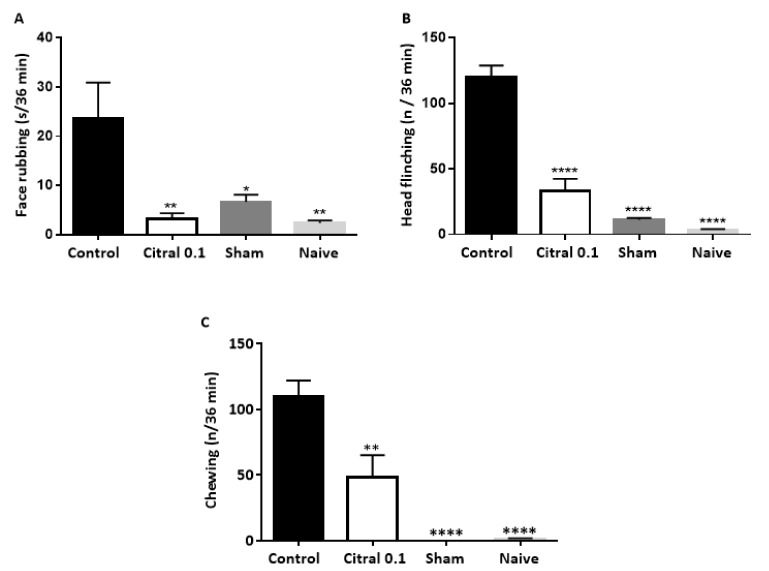
Effect of citral (0.1 mg/Kg) on nociception induced by formalin injection into the temporomandibular joint in rats. Each column represents the mean ± S.E.M (n=6/group) of the time spent in face rubbing (A) or time spent in head flinching (B) and chewing (C). One-way ANOVA with *post-hoc* Tukey's test (*p<0.05; **p<0.01 and ****p<0.0001 *vs *control). Formalin (2.0 %) was injected into the left temporomandibular joint of animals 60 minutes after citral or vehicle (control) administration. Citral and vehicle (control) were administered by gavage.

**Figure 5:**
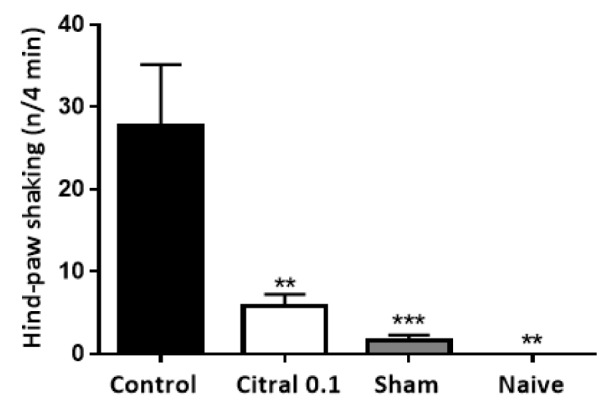
Effect of citral (0.1 mg/Kg) in craniofacial nociception model reflected in mustard oil-induced hind-paw shaking in rats. Each column represents the mean ± S.E.M (n=6/group). Numbers above the bars indicate the percentage of analgesia. One-way ANOVA with *post-hoc* Tukey's test (**p<0.01; ***p 0.001 *vs *control). Mustard oil (20 %) was injected into the left masseter of animals 60 minutes after citral or vehicle (control) administration. Citral and vehicle (control) were administered by gavage.

Injection of formalin into the temporomandibular joint resulted in face rubbing, head flinching and chewing.As shown in Figure 4(Fig. 4), citral (0.1 mg/Kg) reduced the formalin-induced face rubbing, head flinching and chewing (p<0.01, p<0.0001 and p<0.01, respectively). Intramuscular injection of mustard oil into the masseter produced hind-paw shaking. Citral (0.1 mg/Kg) administration resulted in a significant attenuation (p<0.01) of the shaking behavior (Figure 5(Fig. 5)).

**Figure 6:**
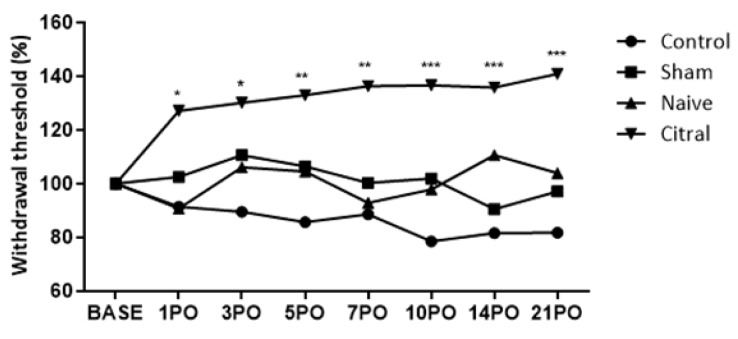
Time course of effect of citral (0.1 mg/Kg) on neuropathic nociception induced by infraorbital nerve transection in rats. PO = Postoperatively. Two-way ANOVA with post-hoc Bonferroni correction (*p<0.05, **p<0.01, ***p<0.001 vs control). Citral and vehicle (control) were administered by gavage. Data are expressed as the mean ± S.E.M. (n=6/group) and compared to control group. Control (BASE 0.000, 1PO 0.081, 3PO - 0.058, 5PO 0.046, 7PO 0.095, 10PO 0.036, 14PO 0.049, 21PO 0.051). Sham (BASE 0.000, 1PO 0.147, 3PO 0.058, 5PO 0.126, 7PO 0.073, 10PO 0.065, 14PO 0.065, 21PO 0.049). Naïve (BASE 0.000, 1PO 0.083, 3PO 0.087, 5PO 0.064, 7PO 0.133, 10PO 0.097, 14PO 0.127, 21PO 0.133). Citral (BASE 0.000, 1PO 0.09579*, 3PO 0.13792*, 5PO 0.14895**, 7PO 0.08561**, 10PO 0.08673***, 14PO 0.10492***, 21PO 0.04622***).

Left IONX produced sustained hypersensitivity to facial mechanical stimulation that was reflected as a reduced head-withdrawal threshold for 21 days. The thresholds in sham-operated and naïve rats did not change (p>0.05). When citral (0.1 mg/Kg) or vehicle (control) was administered at post-operative days 1, 3, 5, 7, 10, 14 and 21, there was a reversal (p<0.05 - p<0.001) produced by citral of the reduced mechanical threshold on post-operative days 3-21 when compared with vehicle control (Figure 6(Fig. 6)).

### Molecular docking findings

**Figure 7:**
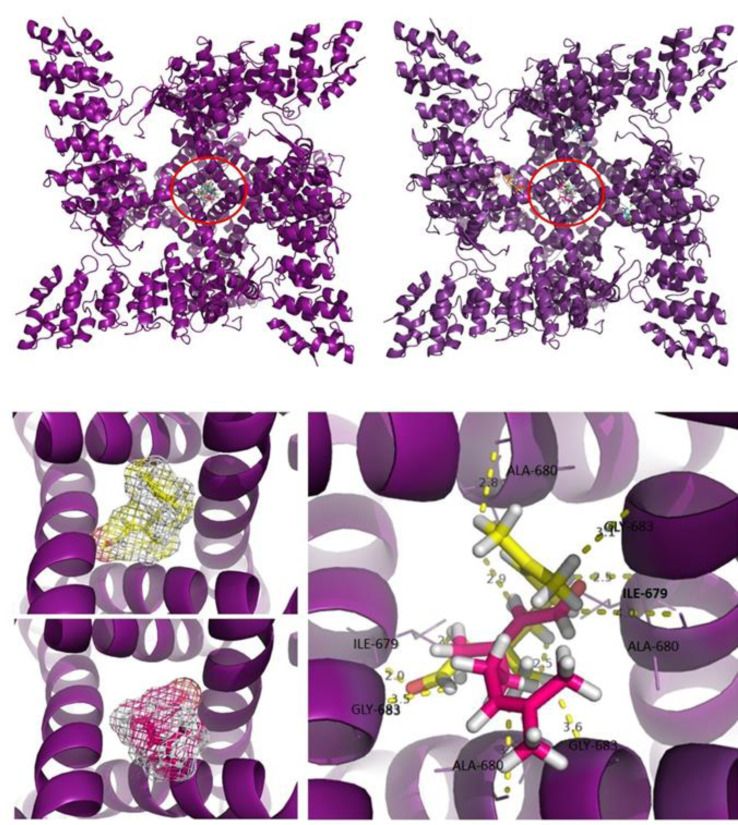
Geranial (upper left panel) showing high specificity for the structural center of the channel, marked in red, since all 10 most energetic clusters overlap in the same place. This is not the case with the neral (upper right panel) isomer, overlapping only 3 of the 10 most energetic clusters. Steric impediment caused by the most energetic geranial cluster (yellow); steric impediment caused by the neral (pink). In addition to providing greater blockage of the center of the channel, the geranial (yellow) also makes chemical bonds with amino acid residues from the 4 subunits, which does not happen with the neral, it provides greater stability to the geranial.

Geranial showed specificity for the channel center because the 10 most stable clusters were all overlapped in the same region (Figure 7(Fig. 7)). Among the clusters of neral, only three were overlapped in the same site, while another six clusters were in adjacent regions, which suggests low specificity and that neral can also interact with sites that do not compromise the channel function (Figure 7(Fig. 7)).

Regarding the most stable cluster (Figure 7(Fig. 7)), 11 chemical bonds were observed, with recruitment of eight amino acid residues (Gly683a, Gly683b, Gly683c, Ile679a, Ile679c, Ala680b, Ala680c, Ala680d), making interactions of at least 2.0 angstroms. Spatial compatibility and blocking of the channel from the ligand socket were also observed at this site (Figure 7(Fig. 7)).

The affinity, specificity and molecular bases of interaction described above are compatible with the energy data, which describe a stabilization like that found in the TRPV1-capsazepine complex (Table 3(Tab. 3)) and illustrate the ability of geranial to interact with this protein *in silico* and *in vivo*.

**Figure 8:**
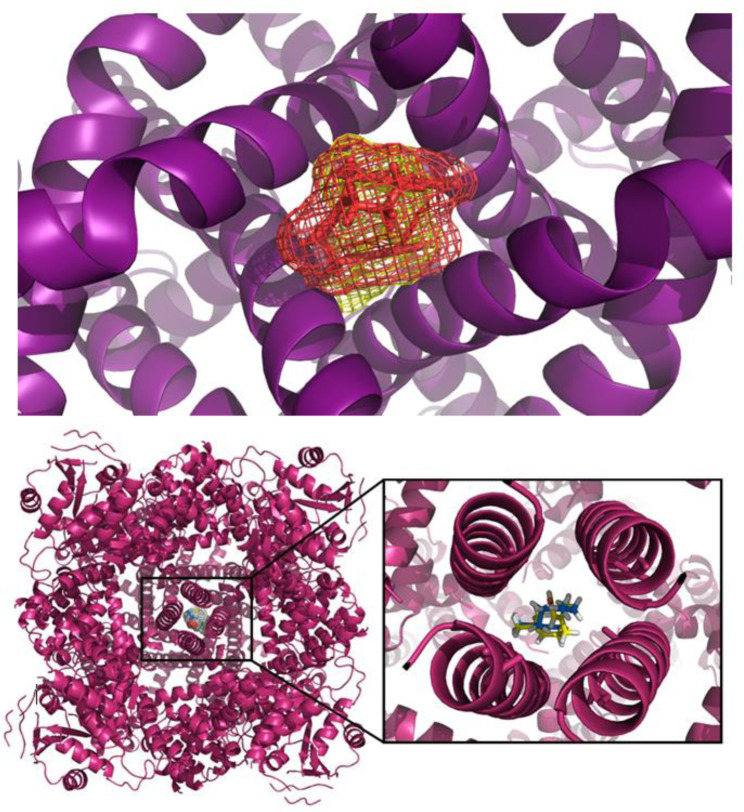
Overlay of the electronic cloud of the geranial (yellow) with capsazepine (red), showing spatial similarities and compatibility in the same site, which supports the action of the geranial resembling that of capsazepine. Neral (blue sticks) and geranial (yellow sticks) isomers interacting specifically with the TRPM8 channel, in the central region, rich in alpha-helix structures.

Many reactive extremities of geranial have been chemically stabilized by hydrogen bonds, as is the case with capsazepine complexation, and despite the difference in molecular mass between geranial and capsazepine (152.23 g/mol and 376.89 g/mol, respectively), it was observed that the electronic cloud of both agents is similar and compatible with the amino acid residues folded in the center of the channel (Figure 8(Fig. 8)). 

Regarding the tests for possible interactions specifically with the TRPM8 channel, it was observed that geranial and neral each had the same affinity for the channel, and had similar specificities, being compatible with the same region (Figure 8(Fig. 8)).

**Figure 9:**
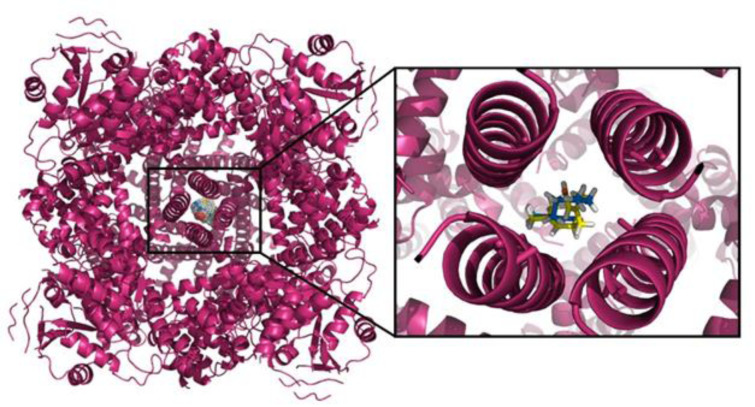
Neral (blue sticks) and geranial (yellow sticks) isomers interacting specifically with the TRPM8 channel, in the central region, rich in alpha-helix structures.

Analyzing these clusters, suggests that the site responsible for the passage of ions is physically blocked by both ligands, compromising the biological function of the channel. The block occurred in a region rich in alpha-helix structures, located in the intracellular portion of the TRPM8 channel (Figure 9(Fig. 9)). The stabilization energy was 166.17 kcal/mol for neral and 149.35 kcal/mol for geranial (Table 4(Tab. 4)). 

### Locomotor activity

Citral did not cause any alteration in the performance of the animals in the rotarod (Table 2(Tab. 2)).

## Discussion

This study evaluated the orofacial antinociceptive potential of citral in animal models of acute or chronic orofacial pain and the possible involvement of TRP channels in its action. In addition to confirming previous studies in rodents of nociceptive behavior produced by application of formalin (Luccarini et al., 2006[37]), cinnamaldehyde (Nomura et al., 2013[51]), menthol (Macpherson et al., 2006[41]) and capsaicin (Pelisser et al., 2002[54]), to the upper lip or by injury of the ION (Kumar et al., 2013[30]), the study provided novel findings that pre-treatment with citral reduced the nociceptive behavior produced in these orofacial pain models, without altering the animals' motor coordination. Citral´s antinociceptive (Nishijima et al., 2014[50]; Mota et al., 2020[48]; Ortiz et al., 2010[53]) and anti-hyperalgesic and anti-inflammatory (Quintans-Júnior et al., 2011[55]; Campos et al., 2019[4]; Gonçalves et al., 2020[15]), effects have already been reported. However, all these studies used much higher citral doses (25 - 300 mg/Kg) than those used in the present study (0.1 - 1.0 mg/Kg). In addition, although the electrophysiological study by Nguyen et al. (2019[49]), suggested that citral might be a modulator of orofacial pain, to our knowledge, the present study is the first to have evaluated and documented the orofacial antinociceptive effect of citral *in vivo*.

Citral, like some other plant compounds, may modulate several different types of TRP ion channels which in the trigeminal system are located in peripheral endings of especially nociceptive afferents, the trigeminal ganglion, and the trigeminal spinal nucleus in the brainstem (Sessle, 2014[61]; Luo et al., 2021[38]; Hossain et al., 2019[19]; Lee et al., 2019[33]). However, the action of citral is quite complex. According to Stotz et al., 2008[62] and others (Ohtsubo et al., 2015[52]; Tomsen et al., 2020[64]), citral may first activate and then produce a long-lasting inhibition of TRP channels such as TRPV1 and TRPM8, while transiently blocking TRPA1 channels, and may involve increases in intracellular calcium concentrations. In contrast, capsaicin, menthol, and both formalin and cinnamaldehyde, are TRP agonists that activate TRPV1, TRPM8, and TRPA1 channels, respectively (Bandell et al., 2004[2]; McNamara et al., 2007[45]) and although menthol is considered a TRPM8 channel agonist (Xu et al., 2020[70]), studies indicate that menthol may also be a TRPA1 activator (Karashima et al., 2007[25]). Our findings are consistent with these various actions in showing that lip or TMJ injections of formalin, cinnamaldehyde, capsaicin or menthol induced nociceptive behaviors, while oral administration of citral significantly induced antinociceptive effects on these nociceptive behaviors. It is noteworthy that whereas citral reduced the nociceptive behaviors induced by the TRPA1 agonists formalin and cinnamaldehyde as well as by the injection into the masseter muscle of mustard oil, another TRPA1 agonist, the antinociceptive effects of citral were not prevented by pretreatment with the TRPA1 antagonist HC-030031 in the cinnamaldehyde test. This may be due to the already existing antinociceptive effect induced by HC-030031 itself, and so an action via the TRPA1 channel cannot be discounted as possibly contributing to the antinociceptive effects of citral. These results suggest that the orofacial antinociceptive effects of citral may be associated with other pathways besides modulation of TRPA1 channels.

The present study did test for other TRP receptor processes that may be involved in citral's effects on orofacial nociception. With the objective of investigating the possible modulation of TRPM8 channels in the effects of citral, mice were treated with repeated applications of menthol which has been shown to produce desensitization of TRPM8 channels (Kuhn et al., 2009[29]; McKemy et al., 2002[44]). We found that this approach eliminated the antinociception produced by citral, suggesting that the citral-induced effects may have involved an action via TRPM8 channels. The possible involvement of TRPM8 channels was supported by the molecular docking study which further indicated the existence of an interaction between citral and TRPM8 channels. The molecular docking study indicated an antagonistic action due to the blocking of the ion passage center and by conformational modification of the TRPM8 structure when entering the protein, thereby compromising the flexibility of the region stabilized by the ligand, and, consequently, its folding. This finding suggests that TRPM8 channel suffers an energy loss when establishing chemical bonds with the geranial and neral isomers of citral. Taken together, these results suggest firstly that the antagonistic action of citral on the TRPM8 channel observed *in vivo* is performed in an equivalent way by both isomers present in the mixture (citral) since they do not present great energy differences, and secondly that affinity and specificity exist for the same connection site. Nonetheless, since the antinociceptive effects of menthol have previously been shown to involve actions on TRPV1 as well as TRPM8 channels (Macpherson et al., 2006[41]; Takaiashi et al., 2016[63]), the reduction of citral's antinociceptive effect on the response to capsaicin when citral was combined with menthol could have involved complex interactions involving the TRPV1 channel. Thus, the present study also investigated the possible modulation of TRPV1 channels in citral's actions. 

As noted above, citral has been reported to have a complex action on the TRPV1 channel; although a prolonged inhibition of the channel is a prominent feature of its action, citral can act as a partial agonist and activate the channel (Stotz et al., 2008[62]; Ohtsubo et al., 2015[52]; Tomsen et al., 2020[64]; Cao et al., 2013[5]; Lu et al., 2005[36]; Vos et al., 2006[65]). Nishijima et al. (2014[50]) for example have demonstrated that citral may act by activating TRPV1 channels at the spinal level, since citral potentiated behaviors indicative of pain produced by intrathecal, but not intraplantar, administration of capsaicin. In the present study, citral reduced the nociceptive behavior induced by the administration of capsaicin to the lip. This effect was not prevented by ruthenium red (non-selective TRP antagonist), consistent with findings by Ohtsubo et al. (2015[52]) in frog sciatic nerves. However, pretreatment of the animals in the present study with capsazepine (a competitive TRPV1 antagonist) completely prevented the antinociceptive effect of citral on the response to capsaicin. This result suggests that citral may interact directly with the TRPV1 channel in the production of antinociception, although the binding site for the TRPV1 channel may be different between citral and capsaicin (Stotz et al., 2008[62]). The molecular docking data also bear on this action of citral since it was observed that both isomers (geranial and neral) present in citral had affinity for the same site, the center of the TRPV1 channel, that is responsible for the passage of electrons, but they presented different specificities. These results suggest that the pharmacological activity observed *in vivo* is performed by the geranial isomer of the applied mixture (i.e., citral). The binding site in which the ligand had the greatest affinity is rich in secondary alpha-helix structures and is the structural center for the passage of ions from the TRPV1 channel (Cao et al., 2013[5]). 

The complex effects of citral could also be related to findings documenting that there are several variants of the TRPV1 channel, including TRPV1b which is a naturally existing inhibitory modulator of TRPV1, and which is not activated by capsaicin and is not affected by capsazepine (Lu et al., 2005[36]; Vos et al., 2006[65]; Luu et al., 2021[39]). Therefore, it is possible in the present study that whereas capsazepine blocked the TRPV1 channel, citral acted not only on TRPV1 to reduce the activity of the TRPV1 channel but it also blocked the TRPV1b variant and thereby reduced its inhibition of the TRPV1 channel, thus making available the TRPV1 channel for capsaicin to produce a nociceptive response even in the presence of capsazepine plus citral. It is also possible that citral itself had some excitatory action on the TRPV1 channel, in line with reports that citral may act in some circumstances as a partial agonist of the TRPV1 channel (Stotz et al., 2008[62]; Ohtsubo et al., 2015[52]; Tomsen et al., 2020[64]). Another possibility is that citral may have acted through an allosteric ligand on the TRPV1 channel (Kaszas et al., 2012[26]; Lebovitz et al., 2012[32]). since it has previously been shown that citral acts as an allosteric modulator of the 5-HT3 receptor (Jarvis et al., 2016[23]) and that the binding sites for capsaicin and citral on the TRPV1 channel are not identical (Stotz et al., 2008[62]). Thus, further studies are warranted to parse out the mechanisms by which TRPV1 channels may contribute to the antinociceptive effects of citral. 

The TRPM3 channel was also investigated in the present study for its possible involvement in citral's effects. This channel is present in sensory neurons of the dorsal root ganglion and trigeminal ganglion according to Krügel et al. (2017[28]) and Yajima et al. (2019[71]) and, according to Vriens et al. (2011[67], 2014[66]) is involved in mechanisms of pain perception and is co-expressed with the TRPV1 channel in some ganglion neurons. Recently, Yajima et al. (2019[71]) pointed out that co-expression of TRPV1 and TRPM3 may be associated with pain transduction in the trigeminal system. Mefenamic acid is known to be a TRPM3 channel antagonist (Klose et al., 2011[27]; Lane Brown et al., 2015[31]) and, like capsazepine, its pre-administration in the present study reversed the antinociceptive effect of citral, suggesting that the terpene may act by modulating TRPM3 and possibly TRPV1 channels. Although mefenamic acid was used as a TRPM3 antagonist in the present study, mefenamic acid has been shown to act also as a partial TRPA1 agonist (Hu et al., 2010[20]) and such an action could have contributed to the only partial reversal by mefenamic acid of citral's antinociceptive effect on the response to the TRPA1 agonist cinnamaldehyde as well as account for the very limited antinociceptive effect of mefenamic acid *per se *on the response to cinnamaldehyde. Mefenamic acid is also known to be a competitive inhibitor of COX-1 and COX-2. However, it is unlikely that its effect on citral's effects involved such a mechanism because of the very limited antinociceptive effect of mefenamic acid *per se*. 

The animals that received transection of the infraorbital nerve (IONX) were found to have prolonged facial hyperalgesia that lasted for many days after IONX, consistent with earlier findings in this trigeminal neuropathic pain model (Kumar et al., 2013[30]; Melo et al. 2019[46]; De Oliveira et al., 2020[8]). The present study also provided novel findings that citral can reverse this IONX-induced orofacial nociceptive behavior throughout the study's observation period (21 days post-operatively). This finding is consistent with a previous report of the efficacy of citral in inhibiting mechanical hyperalgesia induced in a sciatic nerve ligation model of neuropathic pain and in a model of complex regional pain syndrome type-I (Nishijima et al., 2014[50]). However, the doses used were much higher than those used in the present study; and evaluated the antinociceptive effect of citral only until the 15th postoperative day. Here, we have documented that citral at much lower doses had a significant and long-duration antinociceptive effect, lasting at least until the 21st postoperative day. It is also noteworthy that increased gene expression of TRPA1, TRPV1, TRPV2 and TRPM8 (Wu et al., 2016[69]) as well as TRPM3 and TRPV4 (Zhang et al., 2019[72]) has been reported in the spinal trigeminal nucleus of rats subjected to chronic constriction injury of the infraorbital nerve. The antinociceptive effect of citral that we found in the IONX model could possibly be related to its inhibition of TRP channels which are potential targets for the treatment of neuropathic pain (Levine and Alessandri-Haber, 2007[34]; Salat et al., 2013[59]; Marwaha et al., 2016[43]; Sessle, 2021[60]).

The present study has several strengths, such as providing the first documentation that citral compared with vehicle produced antinociceptive effects via TRP channel interactions, but without affecting motor coordination, in both acute inflammatory and chronic (neuropathic) orofacial pain models. There are nonetheless some study limitations that need to be considered. These include the use of only male rats; future studies are needed to determine if citral is equally effective in females or if there is a sex difference in its effects. In addition, future studies could also test if citral is effective also in other models of chronic orofacial pain, such as chronic inflammatory pain, as well as test if its action on the TRPV2 and TRPV3 channels could conceivably be involved (Stotz et al., 2008[62]). Furthermore, the present study was not designed to address where in peripheral tissues or central nervous system citral was acting to exert its antinociceptive effects. Various TRP channels have been documented in orofacial tissues, trigeminal ganglion, and trigeminal spinal nucleus in the brainstem and in analogous components of the spinal nociceptive system (Nishijima et al., 2014[50]; Krügel et al., 2017[28]; Yajima et al., 2019[71]; Vriens et al., 2011[67], 2014[66]; Wu et al., 2016[69]; Zhang et al., 2019[72]; Sessle, 2021[60]). All these are potential sites where citral may be acting and future studies are needed to determine its site(s) of action and other features of its mechanisms in modulating orofacial pain, such as the use of TRPA1- and TRPV1-null mice as well the use of the citral isomers neral and geranial in orofacial pain models.

## Conclusion

These results indicate that the naturally occurring terpene citral attenuates nociceptive behavior in acute and chronic orofacial pain models in rodents, without altering motor coordination. These pre-clinical findings point to the potential clinical usefulness of citral as an analgesic agent in acute and chronic orofacial pain states.

## Declaration

### Funding

The work was supported by CAPES (#053/2014), CNPq (#302319/2019-0), FUNCAP (#BMD-0124-00063.01.00/17), UNIFOR Global Research Fellowship Award (#PS00283112), and Edson Queiroz Foundation (#49/2019).

### CRediT authorship contribution statement

Sacha Aubrey Alves Rodrigues Santos: Investigation, conceptualization, methodology, writing - original draft, review & editing. Marina de Barros Mamede Vidal Damasceno: Methodology, writing - original draft. Francisco Ernani Alves Magalhães: Methodology, writing - original draft. Barry John Sessle: Data analysis and review and editing of draft. Breytiner Amaro de Oliveira: Methodology, writing - original draft. Francisco Lucas Alves Batista: Methodology, writing - original draft. Antônio Eufrásio Vieira-Neto: Methodology, writing - original draft. Adriana Rolim Campos: Conceptualization, methodology, writing - original draft, supervision, project administration, funding acquisition.

### Declaration of competing interest

The authors declare that there are no conflicts of interest.

## Figures and Tables

**Table 1:**
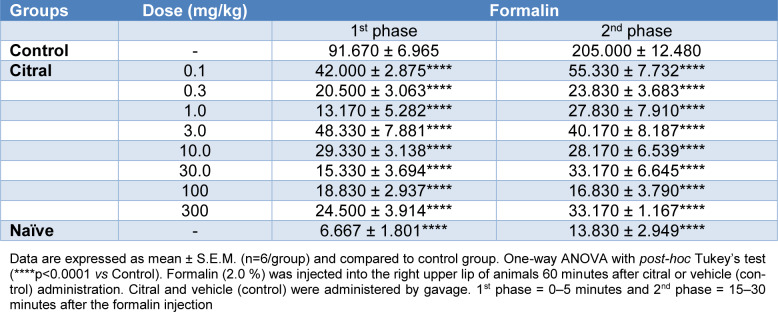
Effect of citral on formalin 1st phase and 2nd phase nociception in mice

**Table 2:**
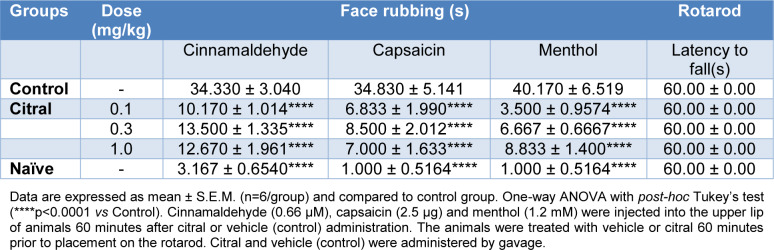
Effect of citral on cinnamaldehyde, capsaicin and menthol nociception in mice

**Table 3:**
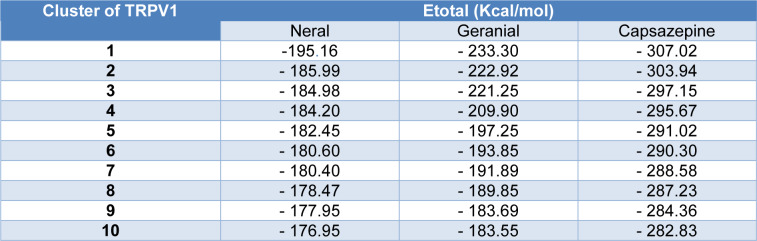
Binding energies between citral isomers and capsazepine against TRPV1 channel (E_total_ - Kcal/mol)

**Table 4:**
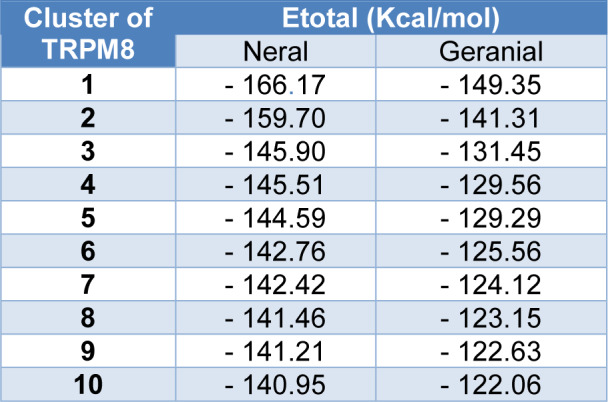
Binding energies between citral isomers and TRPM8 channel (E_total_ - Kcal/mol)

**Figure 1:**
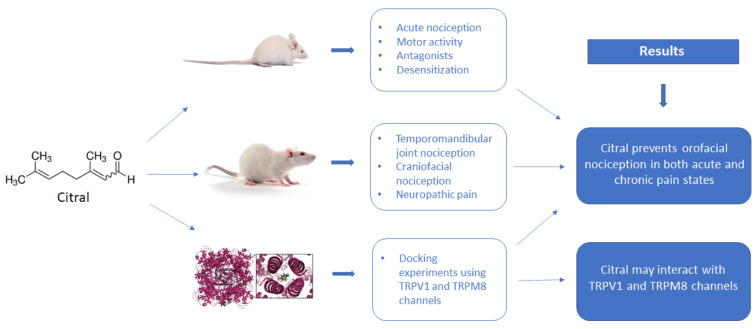
Graphical abstract
